# Floral vasculature and its variation for carpellary supply in *Anthurium* (Araceae, Alismatales)

**DOI:** 10.7717/peerj.2929

**Published:** 2017-01-26

**Authors:** Letícia P. Poli, Lívia G. Temponi, Alessandra I. Coan

**Affiliations:** 1 Departamento de Botânica, Universidade Estadual Paulista “Júlio de Mesquita Filho” - UNESP, Rio Claro, São Paulo, Brazil; 2 Centro de Ciências Biológicas e da Saúde, Universidade Estadual do Oeste do Paraná - UNIOESTE, Cascavel, Paraná, Brazil

**Keywords:** Dorsal bundle, Carpel, Vascular complex, Monocotyledons, Synlateral bundle

## Abstract

**Introduction and Aims:**

*Anthurium* is the largest genus of Araceae, with 950 species distributed in the neotropics. Despite the great diversity of the genus, the knowledge of its floral vasculature is based on observations in only two species, viz. *A. denudatum* and *A. lhotzkyanum*, with remarkable variation in vascular carpellary supply: carpels are either vascularized by ventral bundles alone or by reduced dorsal bundles in addition to the ventral ones. Our main objective is to test this peculiar variation through a detailed anatomical study of the floral vasculature in taxa belonging to some sections of *Anthurium* designated as monophyletic groups in recent phylogenies.

**Methods:**

We compare the floral vasculature of 20 neotropical species belonging to distinct sections of *Anthurium*, using both light and confocal laser scanning microscopies.

**Results:**

The number and position of vascular bundles are constant within the tepals and stamens, regardless of the species and sections studied. However, the gynoecium vasculature exhibits variation between species belonging to the same or different sections. Our results reveal two patterns of vasculature: carpels vascularized by synlateral bundles alone (Pattern A) and carpels vascularized by both dorsal and synlateral bundles (Pattern B). Pattern A is shared by the majority of species studied here and corroborates the previous data in the literature. Pattern B occurs in three species: *A. affine* (*Anthurium* sect*. Pachyneurium* series *Pachyneurium*), *A. obtusum* and *A. scandens* (*Anthurium* sect. *Tetraspermium*), described here for the first time for the genus.

**Conclusions:**

The variation in the supply to the carpels in *Anthurium* is corroborated here. However, our results in addition to those from the available literature suggest the existence of three patterns (A, B and C) of carpellary vasculature. Based on the recent phylogeny of *Anthurium* it is possible to notice that the three patterns of carpellary vasculature occur in representatives of Clade B and deserve to be investigated in a larger number of species. Pattern A could be a plesiomorphy for the genus and the occurrence of dorsal bundles could be a derived character. Our data contributes to the taxonomy and to the understanding of the floral evolution of the largest neotropical genus of Araceae.

## Introduction

Araceae is the largest family among the Alismatales and possesses 125 genera and 3,525 species (Boyce & Croat, 2016), divided into eight principal clades: Aroideae, Gymnostachydoideae, Lasioideae, Lemnoideae, Monsteroideae, Orontioideae, Pothoideae and Zamioculcadoideae (Cabrera et al., 2008; Cusimano et al., 2011). Pothoideae consists of the genera *Pothos* L., *Pothoidium* Schott, *Pedicellarum* M. Hotta and *Anthurium* Schott; the latter is the largest genus of the family, with 950 species (Boyce & Croat, 2016).

Historically, representatives of *Anthurium* were circumscribed in 18–19 sections (Engler, 1905; Croat & Sheffer, 1983) (Table 1), based on vegetative characteristics. The recent phylogenetic study by Carlsen & Croat (2013), based on molecular data, revealed 18 clades, many of which lack a corresponding classification in the sections suggested by Engler (1905) and Croat & Sheffer (1983) (Table 1). However, four of these sections are well represented in Brazil (Boyce & Croat, 2016) and present definite correspondence with clades of the phylogeny.

*Anthurium* sect. *Dactylophyllium* (Schott) Engler emend. Croat & Carlsen (2013) consists of 24 species and corresponds to Clade 3 in the phylogeny of Carlsen & Croat (2013) (Table 1). *Anthurium* sect. *Pachyneurium* Schott encompasses approximately 120 species (Engler, 1905; Croat, 1991), divided into two series: *A*. series *Pachyneurium* (Schott) Croat and *A*. series *Multinervia* Croat, which correspond to Clades 9 and 12 and to Clade 11, respectively, in the phylogeny of Carlsen & Croat (2013) (Table 1). *Anthurium* sect. *Tetraspermium* Schott is a reduced group, formed by only four species (Engler, 1905); the section is designated as monophyletic and corresponds to Clade 5 in the phylogeny of Carlsen & Croat (2013) (Table 1). Finally, *Anthurium* sect. *Urospadix*, with approximately 100 species (Engler, 1905; Govaerts et al., 2002), is the most artificial section of the genus (Croat & Sheffer, 1983) and has been designated as a group that needs redefining, in which the endemic species of Brazil form one monophyletic group and correspond to Clade 1 in the phylogeny of Carlsen & Croat (2013) (Table 1).

**Table 1:** Comparison of early classification systems with the recent phylogeny of *Anthurium*.

Sections *sensu* Engler (1905)	Sections *sensu* Croat & Sheffer (1983)	Clades or groups *sensu* Carlsen & Croat (2013)
*Belolonchium* Schott	*Belolonchium* Schott emend Engler	Clades 4, 14 and 16
*Calomystrium* Schott	*Calomystrium* Schott emend Engler	Clade 13
*Cardiolonchium* Schott	*Cardiolonchium*	Clades 10, 12 and 16
*Chamaerepium* Schott	*Chamaerepium*	Clade 1
	*Dactylophyllium* Schott	Clade 3
*Digitinervium* Sodiro	*Digitinervium*	Clade 7
*Episeiostenium* Schott	*Episeiostenium*	Clade 2
*Gymnopodium* Engler	*Gymnopodium*	Not sampled
*Leptanthurium* Schott	*Leptanthurium*	Clade 8
*Oxycarpium* Schott	*Oxycarpium*	Clades 8, 10, and 16
*Pachyneurium* Schott	*Pachyneurium*	Clades 9, 11 and 12
*Polyneurium* Engler	*Polyneurium*	Clades 10 and 15
*Polyphyllium* Engler	*Polyphyllium*	Clade A
*Porphyrochitonium* Schott	*Porphyrochitonium*	Clades 6 and 7
*Schizoplacium* Schott	*Schizoplacium*	Clades 3, 14, and 16
*Semaeophyllium* Schott	*Semaeophyllium*	Clade 14
*Tetraspermium* Schott	*Tetraspermium*	Clade 5
*Urospadix* Engler	*Urospadix*	Clades 1 and 2
*Xialophyllium* Schott	*Xialophyllium*	Clade 15

Floral anatomical studies in Araceae indicate variation in the gynoecium vasculature and point out its applicability to the understanding of certain evolutionary aspects (e.g., Eyde, Nicolson & Sherwin, 1967; Buzgo & Endress, 2000; Buzgo, 2001). An example is the reduction of dorsal vascular bundles in the gynoecium of *Schismatoglottis* Zoll. & Moritzi, designated by Hotta (1971) as a characteristic associated with variation in the number of carpels and ovules and with sub-basal placentation. Also emphasized is the identification of additional vascular bundles supplying the carpels, leading to the confirmation of pseudomonomery in *Calla* L. (Barabé & Labrecque, 1983), *Lysichiton* Schott (Barabé & Labrecque, 1984), *Orontium* L. (Barabé & Labrecque, 1985) and *Symplocarpus* Salisb. (Barabé, Forget & Chrétien, 1986). In studies of *Monstera* Adans (Barabé & Chrétien, 1985) and *Spathiphyllum* Schott (Barabé & Chrétien, 1986), the variation in the pattern of gynoecium vasculature was related to the size and volume of the carpels.

Despite these studies on floral anatomy and vasculature in Araceae, there is still a lack of information with regard to the genus *Anthurium*, in which all knowledge is limited to only two species: *Anthurium denudatum* Engler (*A*. sect. *Belolonchium* Schott) (Carvajal, 1977) and *A. lhotzkyanum* Schott (*A*. sect. *Urospadix*) (Barabé, Forget & Chrétien, 1984). In *A. denudatum*, Carvajal (1977) verified the occurrence of underdeveloped dorsal bundles restricted to the gynoecium base, with each carpel vascularized solely by ventral bundles. In *A. lhotzkyanum*, synonymous of *A*. *augustinum* K. Koch & Lauche (Cardozo et al., 2014), Barabé, Forget & Chrétien (1984) reported only ventral complexes and placental traces, which diverge from four complexes at the floral base. Thus, an infrageneric variation has been observed in relation to the carpellary vasculature as well as previously indicated for other floral characteristics (Carvajal, 1977; Higaki, Rasmussen & Carpenter, 1984; Carvell, 1989; Poli, Temponi & Coan, 2012; Poli, Temponi & Coan, 2015). These characteristics are contributing to new data toward the understanding of the floral morphology of the genus and may help in the still widely debated infrageneric classification.

The study of floral vasculature in more representatives of *Anthurium*, specifically in traditionally recognized groups such as the four previously described, is expected to point out new anatomical data that may be used in their delimitation. Thus, the present study proposes a comparative analysis of floral vasculature of *Anthurium* species, with emphasis on gynoecial aspects, seeking to answer the following questions: (1) Are the patterns of carpellary vasculature previously reported for *Anthurium* found in the taxa analyzed here; (2) Do the floral vascular characteristics aid in the delimitation of *Anthurium* sect. *Dactylophyllium*, *A*. sect. *Pachyneurium* series *Pachyneurium*, *A*. sect. *Tetraspermium* and *A*. sect. *Urospadix*?

## Materials and Methods

**Figure 1:**
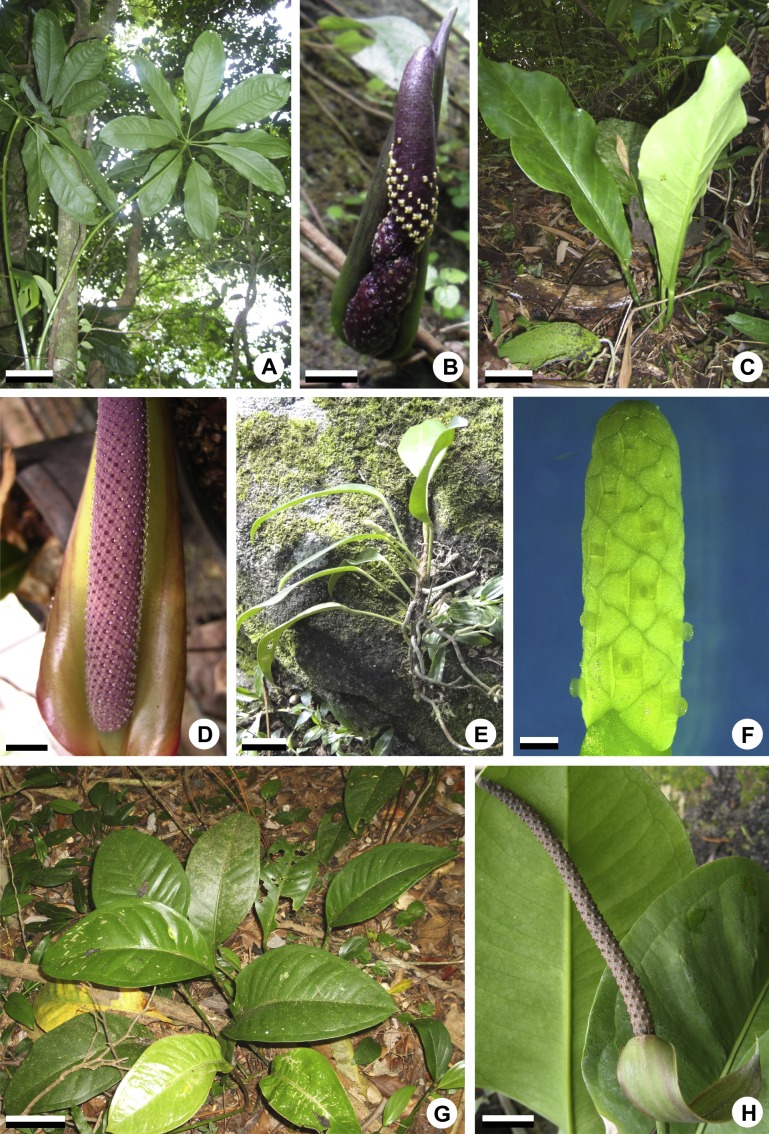
General aspects of habit and inflorescences of some representatives of *Anthurium*. (A, B) Habit (A) of *A. pentaphyllum* (*A.* sect. *Dactylophyllium*) and its inflorescence (B) with most flowers at male anthesis. (C, D) Habit (C) of *A. solitarium* (*A.* sect. *Pachyneurium* series *Pachyneurium*) and its inflorescence (D) with flowers at female anthesis. (E, F) Habit (E) of *A. scandens* (*A.* sect. *Tetraspermium*) and detail of its inflorescence (F) with most flowers at female anthesis. (G, H). Habit (G) of *A. miquelianum* (*A.* sect. *Urospadix*) and its inflorescence (H) with flowers in post-female stage. Scale bars: A–D, G, H = 15 mm; E = 30 mm; F = 1 mm.

Neotropical representatives of four traditional sections of *Anthurium*, mainly distributed in Brazil, were selected for the present study (Table 2; Fig. 1). Vouchers were deposited at the herbaria HRCB, RB, SPF, UB, UFP, VIC (acronyms according to *Index Herbariorum*, Thiers, 2016) and at the herbarium of the Universidade Estadual do Oeste do Paraná (Cascavel, Paraná, Brazil). The material examined is listed in Table 2, based on the following field permits: SisBio permanent permit no. 40816-2 (April 2011 to present) to LP Poli; SisBio collecting permits no. 28776 (June 2011–May 2014) and 28686 (June 2011–May 2014) to LG Temponi.

**Table 2:** Specimens of *Anthurium* examined and respective collections. Infrageneric classification sensu Croat & Sheffer (1983) and clades based on Carlsen & Croat (2013).

Section/Species	Clade	Location	Collector number/herbarium
***Anthurium*** **sect.** ***Dactylophyllium*** **(Schott)** **Engler emend. Croat & Carlsen**	Clade 3		
*Anthurium pentaphyllum* (Aubl.) G.Don		Brazil, Minas Gerais, Marliéria	LG Temponi et al. 119 (VIC)
		Brazil, Bahia, Ipiaú	LG Temponi et al. 344 (SPF)
***Anthurium*** **sect.** ***Pachyneurium*** **series** ***Pachyneurium*** **(Schott) Croat**	Clades 9 and 12		
*Anthurium affine* Schott		Brazil, Pernambuco, Altinho	M Sobral-Leite et al. 946 (UFP)
		Brazil, Pernambuco, Caruaru-Agrestina	P Gomes et al. 256 (UFP)
*Anthurium atropurpureum* var. *arenicola* Croat		Brazil, Amazonas, Manaus	LP Poli & JAC Silva 51 (HRCB)
*Anthurium lindmanianum* Engler		Brazil, Mato Grosso, Chapada dos Guimarães	MN Saka et al. 539 (HRCB)
*Anthurium solitarium* Schott		Brazil, Rio de Janeiro,Teresópolis	LG Temponi et al. 983 (UNOP)
		Brazil, Rio de Janeiro, Nova Iguaçu	LG Temponi et al. 1063 (UNOP)
***Anthurium*** **sect.** ***Tetraspermium*** **Schott**	Clade 5		
*Anthurium obtusum* (Engler) Grayum		Unknown	Horto Botânico da UCB (Cv), number 00093
*Anthurium scandens* (Aubl.) Engler		Brazil, Rio de Janeiro,Teresópolis	LG Temponi et al. 973 (UNOP)
		Brazil, São Paulo, São Miguel Arcanjo	PLR Moraes et al. 3371 (HRCB)
***Anthurium*** **sect.** ***Urospadix*** **Engler**	Clade 1		
*Anthurium acutum* N.E.Br		Brazil, Santa Catarina, Ilhota	EG Gonçalves et al. 320 (UB)
*Anthurium augustinum* K. Koch & Lauche		Brazil, Rio de Janeiro,Teresópolis	LG Temponi et al. 974 (UNOP)
		Brazil, Rio de Janeiro, Nova Iguaçu	LG Temponi et al. 1051 (UNOP)
*Anthurium comtum* Schott		Brazil, Rio de Janeiro, JBRJ (Cv)	MN Coelho 1402 (RB)
*Anthurium coriaceum* G.Don		Brazil, Rio de Janeiro, Niterói	LG Temponi et al. 297 (SPF)
*Anthurium gladiifolium* Schott		Brazil, Minas Gerais, Salto da Divisa	LG Temponi et al. 272 (SPF)
*Anthurium loefgrenii* Engler		Brazil, Paraná, Paranaguá	LC Ferneda Rocha 239 (UNOP)
*Anthurium longipes* N.E.Br.		Brazil, Bahia, Itacaré	LG Temponi et al. 339 (SPF)
*Anthurium minarum* Sakur. & Mayo		Brazil, São Paulo, Cruzeiro	LG Temponi et al. 366 (SPF)
*Anthurium miquelianum* C. Koch & Augustin		Brazil, São Paulo, Iguape	LP Poli et al. 39 (HRCB)
*Anthurium organense* Engler		Brazil, Rio de Janeiro, Rio de Janeiro	LG Temponi et al. 347 (SPF)
*Anthurium parasiticum* (Vell.) Stellfeld		Brazil, Rio de Janeiro, Rio de Janeiro	LP Poli et al. 40 (HRCB)
		Brazil, Rio de Janeiro, Rio de Janeiro	LG Temponi et al. 413 (SPF)
*Anthurium parvum* N.E.Br		Brazil, Rio de Janeiro, Teresópolis	LG Temponi et al. 975 (UNOP)
*Anthurium sellowianum* Kunth		Brazil, Rio de Janeiro, Teresópolis	LG Temponi et al. 977 (UNOP)
		Brazil, São Paulo, Iguape	LG Temponi et al. 993 (HRCB)

**Notes.**

Cv cultivated JBRJ Jardim Botânico do Rio de Janeiro UCB Universidade Católica de Brasília

**Figure 2:**
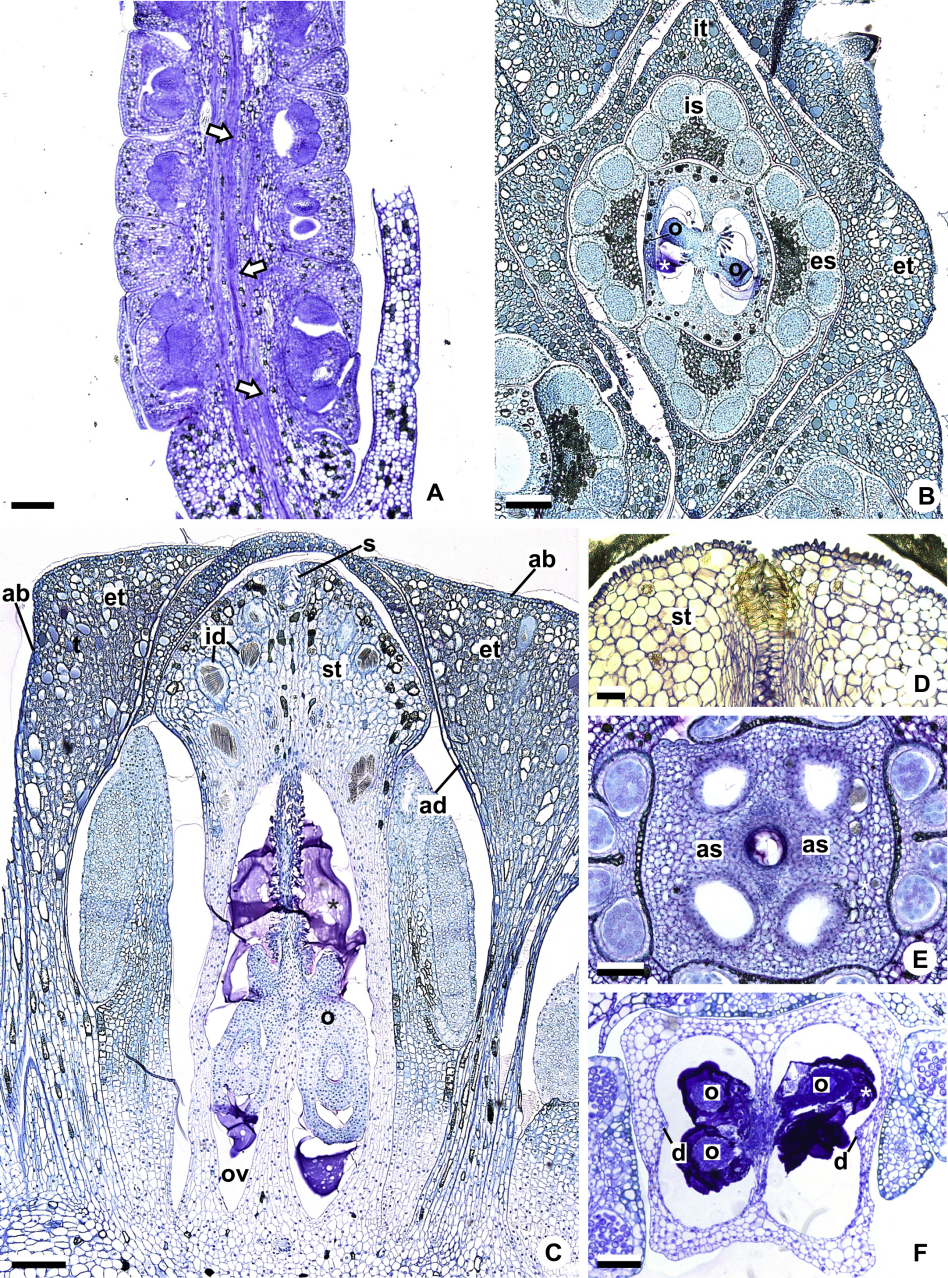
Floral anatomical aspects of species of *Anthurium*, based on longitudinal (A, C, D) or transverse sections (B, E, F). (A) Developing spadix of *A. parvum* showing vasculature in its central axis (arrows). (B) Flower at female anthesis of *A. parvum* showing external and internal stamen and tepal whorls, and bicarpellary gynoecium. (C) Flower at female anthesis of *A. affine* showing gynoecium differentiated into stigma, style and ovary. (D) Detail of style at post-female anthesis of *A. pentaphyllum*. (E) Developing style of *A. scandens*, at the height of the apical septum. (F) Ovary of *A. scandens* with two ovules per locule, showing mucilage and dorsal carpellary bundles. ab, abaxial surface of tepals; ad, adaxial surface of tepals; as, apical septum; d, dorsal carpellary bundle; es, external stamen; et, external tepal; id, crystalliferous idioblasts; is, internal stamen; it, internal tepal; o, ovule; ov, ovary; s, stigma; st, style; *, mucilage. Scale bars: A–C = 200 µm; D–F = 100 µm.

At least two samples of inflorescences from different specimens were analyzed, whenever possible from different localities (Table 2). The inflorescences were collected at different stages of development and fixed in Transeau solution (Bicudo & Menezes, 2006) or FAA 50 (Johansen, 1940).

For the study using light microscopy (LM), samples were dehydrated through a n-butyl alcohol series (Feder & O’Brien, 1968), embedded in 2-hydroxyethyl methacrylate (Leica Historesin Embedding Kit), and sectioned at 7–10 µm on a rotatory microtome (Leica). The anatomical sections were stained with periodic acid Schiff (PAS reaction) and 0.05% Toluidine blue O in 0.1 M sodium phosphate buffer (pH 6.8) (Feder & O’Brien, 1968), or only with 0.05% Toluidine blue O in 0.1 M sodium phosphate buffer (pH 6.8) (O’Brien, Feder & McCully, 1965), and mounted in Entellan (Merck). Samples of gynoecia were also individualized and cleared using the technique of Shobe & Lersten (1967). The results were documented in photomicrographs obtained with the image digitization program LAS (Leica Application Suite v. 4.0; Leica), using an image capture apparatus (DFC-450, Leica) attached to the microscope (DM 4000B, Leica).

For study using confocal laser scanning microscopy (CLSM), samples were embedded in polyethylene glycol (PEG 1500) (Gerlach, 1984), and sectioned at 15–20 µm on a rotatory microtome (Leica). For observations, confocal laser scanning microscope (Leica TCS SP5 II) was used.

Diagrams were produced using CorelDRAW X7 (Corel Corporation) software.

## Results

The results are presented in two main topics: “Floral organography” and “Floral vasculature.” In the first topic a single floral description is presented for all species herein studied because they generally share many anatomical aspects; differences, when present, are emphasized throughout the text. The second topic includes the description of the floral vascular supply in *Anthurium* and is divided into two subtopics: “Pattern A: Carpels vascularized by only synlateral bundles,” and “Pattern B: Carpels vascularized by synlateral and dorsal bundles.”

### Floral organography

The flowers of *Anthurium* are sessile (Fig. 2A) and are arranged spirally along the spadix (Figs. 1B, 1D, 1F and 1H). The flowers are bisexual and dimerous, possessing two external tepals, two internal tepals, two external stamens, two internal stamens and a bicarpellary gynoecium (Figs. 2B and 2C).

All species studied are protogynous; thus, the majority of the anatomical description of the sterile floral parts corresponds to the pistillate stage (Figs. 1D and 1F).

The tepals are free, with a cucullate shape (Figs. 2B and 2C) due to the congested disposition of the flowers on the spadix. Of the external tepals, around two thirds of their adaxial surface come into contact with the external stamens, and the upper third is in contact with the apical portion of the internal tepals (Figs. 2B and 2C); about two thirds of the abaxial surface come into contact with the same region of the adjacent flower, and the upper third is oriented toward the environment (Fig. 2C).

The internal tepals have about two thirds of their adaxial surface in contact with the internal stamens, and the upper third is in contact with the style and stigma (Figs. 2B and 2C); about two thirds of their abaxial surface are found in contact with the same region of the adjacent flower, and the upper third is partly covered by the external tepals and partly oriented toward the environment (Figs. 2B and 2C).

The stamens of both whorls are free (Figs. 2B and 2C), with filaments formed by epidermis composed of isodiametric cells, with phenolic accumulation and parenchymatous mesophyll (Fig. 2C). The connective is rich in phenolic idioblasts (Fig. 2B). The anthers are bithecal and tetrasporangiate (Fig. 2B).

The gynoecium is differentiated into stigma, style and ovary (Fig. 2C). The stigma is composed of secretory trichomes (Fig. 2C). The style is formed by external epidermis composed of isodiametric cells (Fig. 2C) (papillate only in *A. pentaphyllum* (*A*. sect. *Dactylophyllium*)) (Fig. 2D), parenchymatous mesophyll containing phenolic and crystal idioblasts, and a single layered internal epidermis.

The ovary is superior and bicarpellary, with a septum separating the locules (Figs. 2B and 2C). Only in *A. obtusum* and *A. scandens* (Fig. 2E) (*A*. sect. *Tetraspermium*) does the formation of an apical septum occur, situated between the opening of the stylar canal and the ovarian locules. The locules are filled with mucilage (Figs. 2B, 2C and 2F). The placenta is axial and forms a single ovule per locule (Figs. 2B and 2C), except in *A. obtusum* and *A. scandens* (Fig. 2F) (*A*. sect. *Tetraspermium*), in which two ovules form per locule.

### Floral vasculature

Based on our sample of 20 species of *Anthurium* from four distinct sections (Table 2), two patterns of floral vasculature were observed, related primarily to the carpellary supply.

These patterns are illustrated through diagrams, based on median longitudinal sections (Figs. 3A and 3B) and on transverse sections at different heights of the flower (Figs. 4 and 5), and photomicrographs (Figs. 6–11).

**Figure 3:**
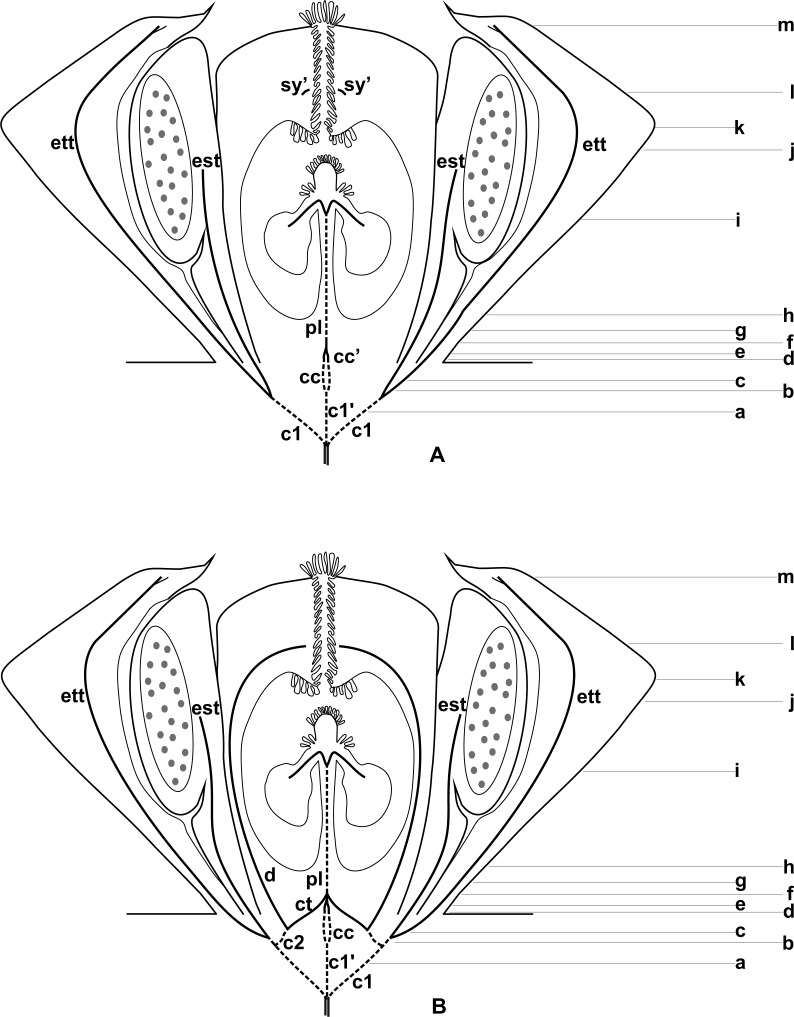
Diagrams of median longitudinal sections of flowers at female anthesis of *Anthurium*. (A) *Anthurium. sellowianum* (*A.* sect. *Urospadix*) and (B) *A. scandens* (*A.* sect. *Tetraspermium*), showing the respective positions (a–m) of transverse sections displayed in the Figs. 4 and 5. c1, external vascular complex; c1’, internal vascular complex; c2, vascular complex originated from c1; cc, carpellary complex; cc’, vascular trace; ct, carpellary trace; d, dorsal carpellary bundle; est, external stamen trace; ett, external tepal trace; pl, placental complex; sy’, branching of synlateral bundle.

**Figure 4:**
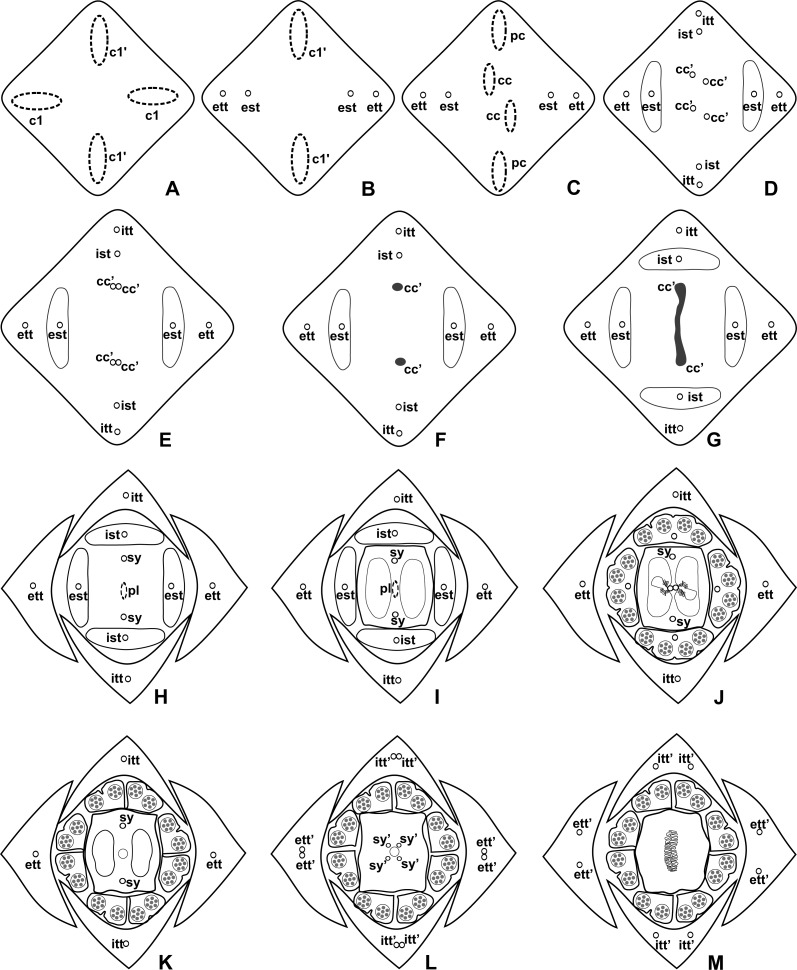
Diagrams of serial transverse sections from the base (A) to the apex (M) of the flower at female anthesis of *A. sellowianum* (*A.* sect. *Urospadix*). c1, external vascular complex; c1’, internal vascular complex; cc, carpellary complex; cc’, vascular trace; est, external stamen trace; ett, external tepal trace; ett’, branching of external tepal trace; ist, internal stamen trace; itt, internal tepal trace; itt’, branching of internal tepal trace; pc, peripheral complex; pl, placental complex; sy, synlateral carpellary bundle; sy’, branching of synlateral bundle.

**Figure 5:**
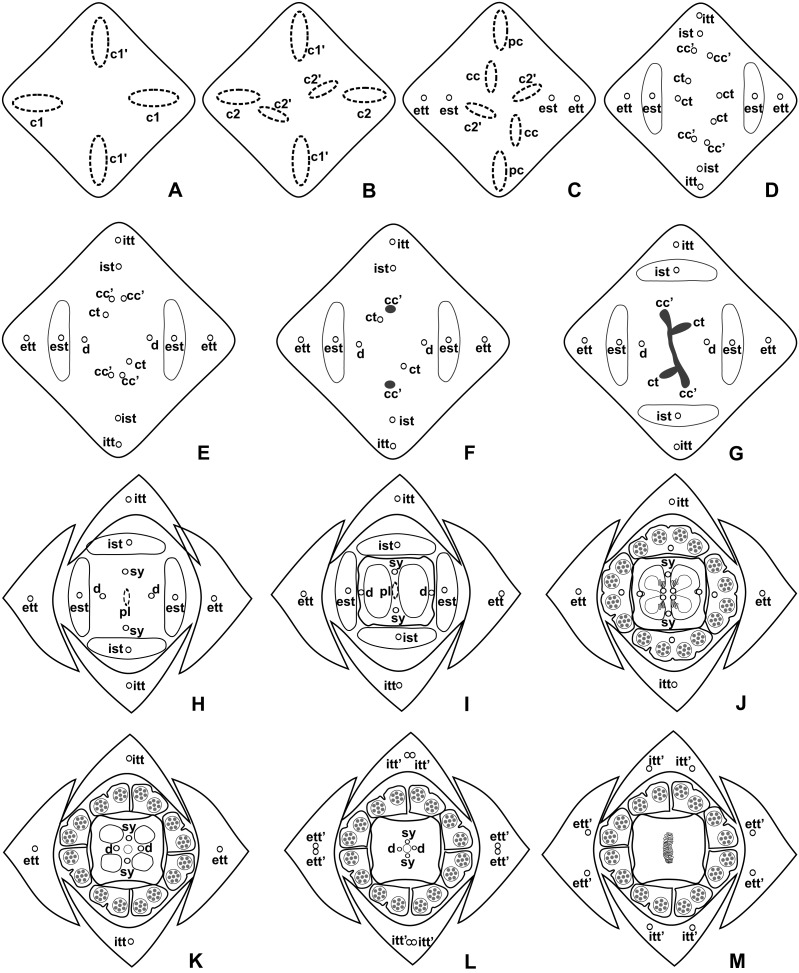
Diagrams of serial transverse sections from the base (A) to the apex (M) of the flower at female anthesis of de *A. scandens* (*A.* sect. *Tetraspermium*). c1, external vascular complex; c1’, internal vascular complex; c2 and c2’, vascular complexes originated from c1; cc, carpellary complex; cc’, vascular trace; ct, carpellary trace; d, dorsal carpellary bundle; est, external stamen trace; ett, external tepal trace; ett’, branching of external tepal trace; ist, internal stamen trace; itt, internal tepal trace; itt’, branching of internal tepal trace; pc, peripheral complex; pl, placental complex; sy, synlateral carpellary bundle.

**Figure 6:**
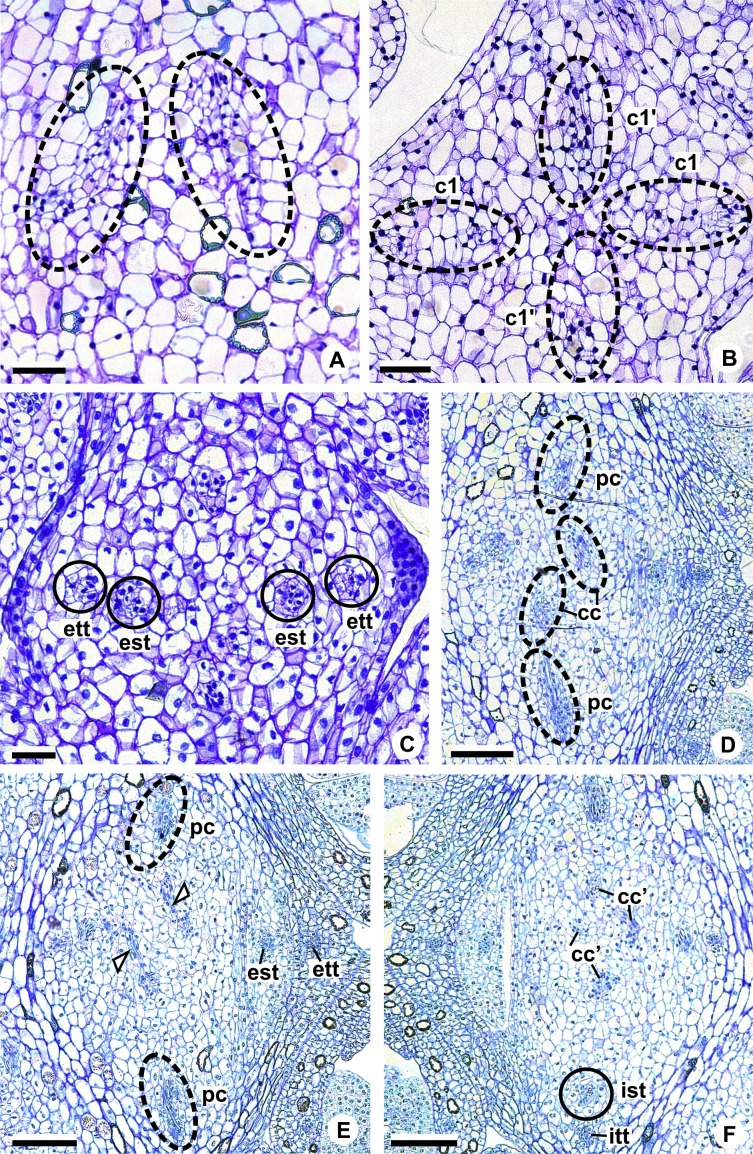
Floral vasculature of species of *Anthurium* sect. *Urospadix*, in transverse sections of developing flowers. (A) Axis of the spadix of *A. gladiifolium* with two vascular complexes. (B) Floral base of *A. gladiifolium* with four complexes. (C) Floral base of *A. loefgrenii* showing both external stamen and tepal traces. (D) Floral base of *A. augustinum* showing peripheral and carpellary complexes. (E) Floral base of *A. augustinum* showing each carpellary complexes diverging into two traces (arrowheads). (F) Floral base of *A. augustinum* showing four vascular traces (cc’). c1, external vascular complex; c1’, internal vascular complex; cc, carpellary complex; cc’, vascular trace; est, external stamen trace; ett, external tepal trace; ist, internal stamen trace; itt, internal tepal trace; pc, peripheral complex. Scale bars: A–C = 50 µm; D–F = 200 µm.

### Pattern A: carpels vascularized by only synlateral bundles (Figs. 3A and 4)

Pattern A was observed in the majority of the studied species, which correspond to the representatives of *A*. sect. *Dactylophyllium*, *A*. sect. *Urospadix*, and three out of four studied species of *A*. sect. *Pachyneurium* series *Pachyneurium*.

The vasculature of the axis of the spadix diverges to the flowers (Fig. 2A—white arrows), branching into two (Fig. 6A) and, posteriorly, four (Figs. 4A and 6B) vascular complexes that are visible at the base of the flower. Two complexes vascularize the external tepal and stamen whorls (Figs. 4A and 6B), referred to here as external complexes (c1); the other two vascularize the internal tepal and stamen whorls and the carpels (Figs. 4A and 6B), referred to here as the internal complexes (c1’).

Initially, each of the two external complexes (c1) (Fig. 4A) diverges into two traces that vascularize the external tepals and stamens (Figs. 4B and 6C). The trace of the external tepal (ett) remains singular for almost the entire length of this organ (Figs. 4C–4K and 6C–6F), except in the upper third, where it branches into two (ett’) (Figs. 4L and 4M). The trace of the external stamen (est) remains singular up to the connective (Figs. 4C–4J).

**Figure 7:**
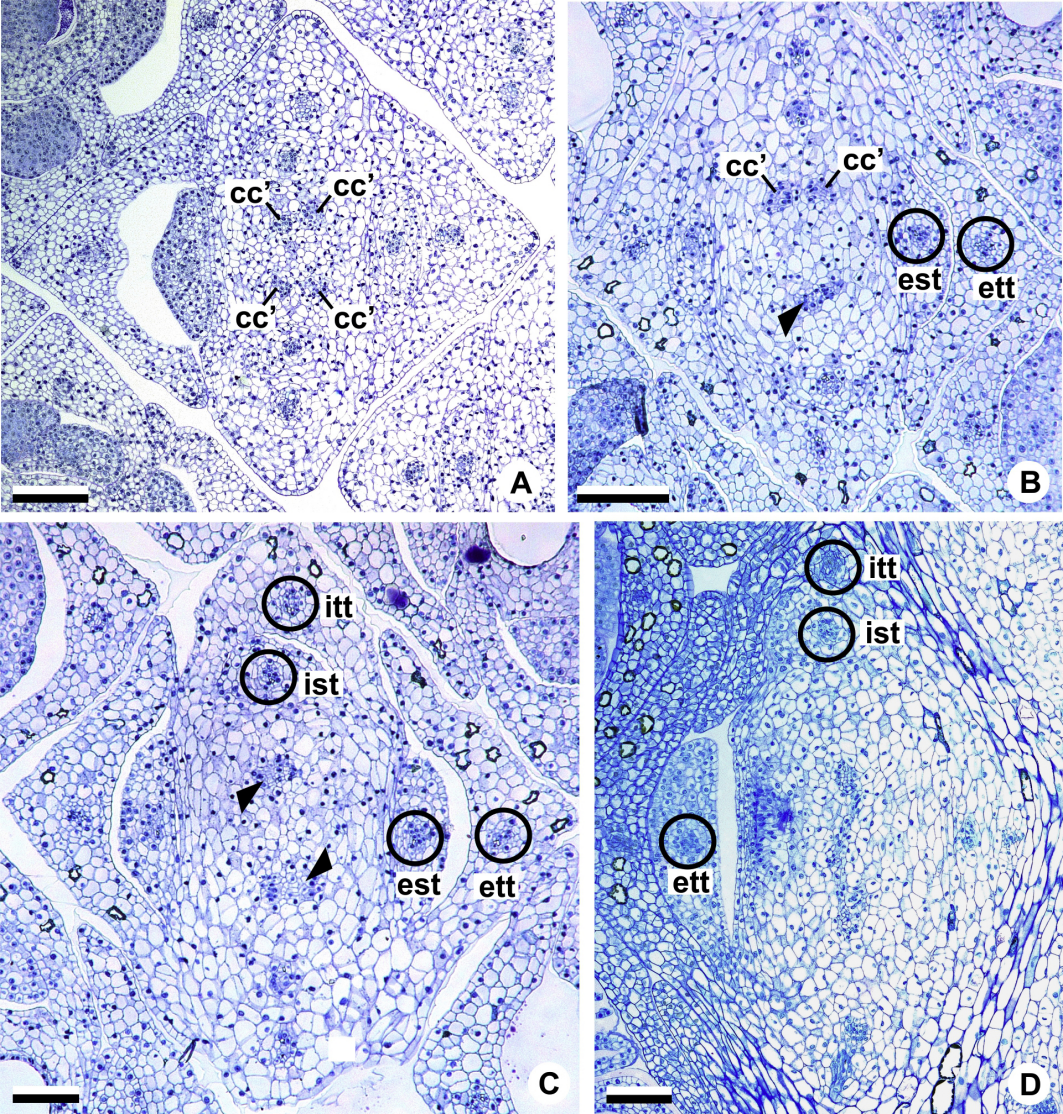
Floral vasculature of species of *A.* sect. *Pachyneurium* series *Pachyneurium* (A–C) and *A*. sect. *Urospadix* (D), in transverse sections of developing flowers. (A) Floral base of *A. solitarium* with vascular traces. (B, C) Successive sections of floral base of *A. atropurpureum* var. *arenicola* showing the rearrangement of carpellary traces (arrowheads), and vascular traces of both whorls of stamens and tepals. (D) Floral base of *A. augustinum*. cc’, vascular trace; est, external stamen trace; ett, external tepal trace; ist, internal stamen trace; itt, internal tepal trace. Scale bars: A, B, D = 150 µm; C = 100 µm.

Next, each of the two internal complexes (c1’) (Figs. 4A and 4B) develops two other, smaller complexes: one peripheral (pc) and one carpellary (cc) (Figs. 4C and 6D). Each peripheral complex (pc) (Fig. 4C) diverges into two traces: one to the internal tepal and the other to the internal stamen (Figs. 4D–4G and 6D–6F). The trace of the internal tepal (itt) remains singular for almost its entire length (Figs. 4E–4K and 7A–7C), except in the upper third, where it branches into two (itt’) (Figs. 4L and 4M). The trace of the internal stamen (ist) extends to the connective (Figs. 4D–4J).

**Figure 8:**
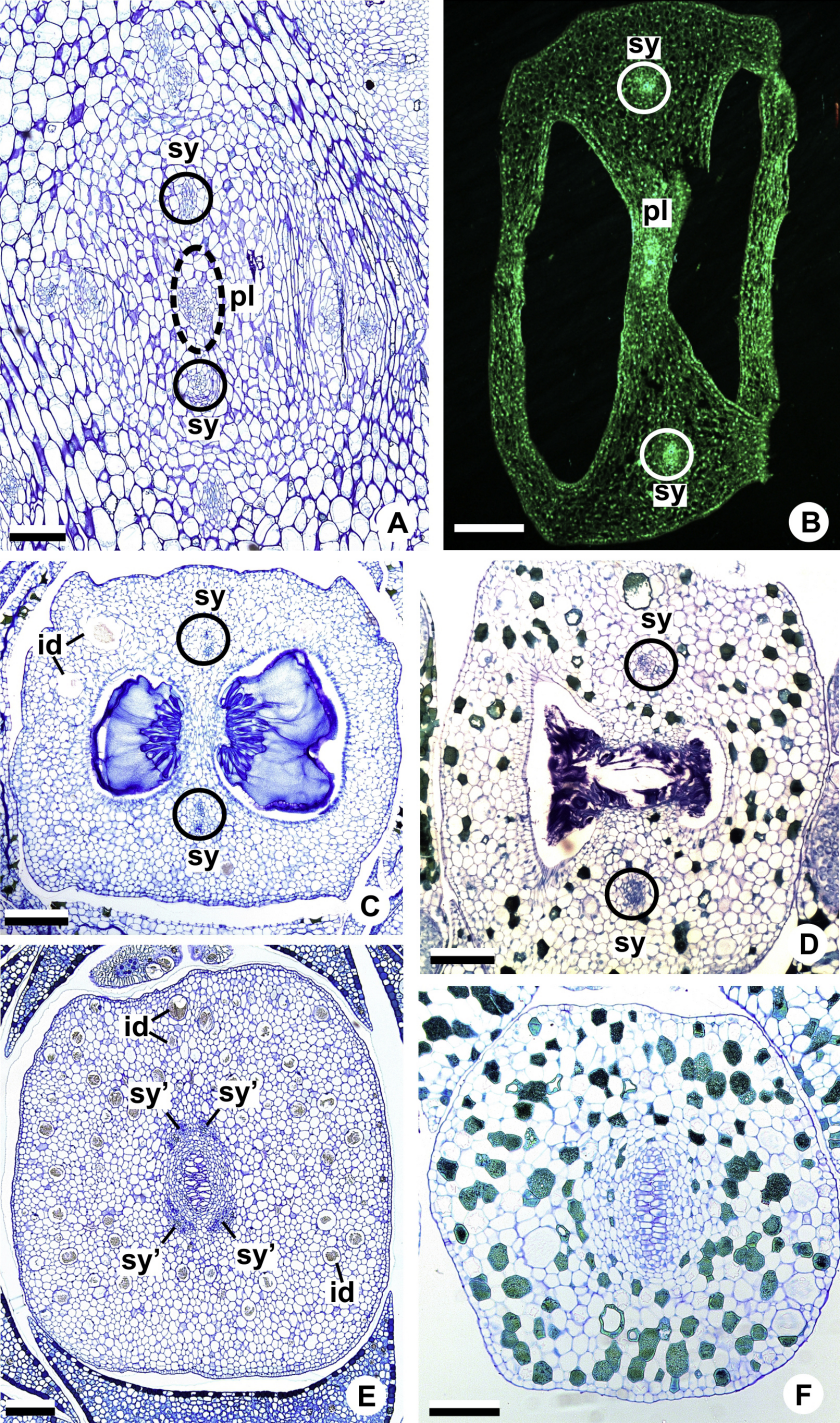
Floral vasculature of species of *A*. sect. *Urospadix* (A, D, F), *A.* sect. *Pachyneurium* series *Pachyneurium* (B, E) and *A*. sect. *Dactylophyllium* (C), in transverse sections of flowers at female anthesis. (A) Floral base of *A. sellowianum* with synlateral carpellary bundle and placental complex. (B) Ovarian base, in confocal laser scanning microscopy (CLSM) of *A. solitarium* with synlateral carpellary bundles and placental complex. (C) Ovarian base, at the height of secretory trichomes of ovarian septum, of *A. solitarium* showing placental complex and synlateral bundle. (D) Style of *A. longipes*, at the height where the stylar canal opens in the ovary, showing two synlateral bundles. (E) Style of *A. solitarium* showing four bundles adjacent to stylar canal, at the upper third of the flower. (F) Stigma of *A. comtum*. id, crystalliferous idioblasts; pl, placental complex; sy, synlateral carpellary bundle; sy’, branching of synlateral bundle. Scale bars: A, B, E = 200 µm; C = 400 µm; D, F = 150 µm.

Each of the two carpellary complexes (cc) (Figs. 4C and 6D) diverges into two traces (cc’) (Figs. 4D, 6E—arrowheads, 6F), which merge (Figs. 4E–4F and 7A–7C—arrowheads) and then split (Figs. 4G and 7D) into two more peripheral bundles (sy) (Figs. 4H and 8A), and a placental complex (pl) (Figs. 4H and 8A). These peripheral bundles and placental complex are heterocarpellary and they are here denominated synlateral bundles. Each synlateral bundle, located between the margin of the two carpels and the ovarian wall (Fig. 8B), extends up to the height of the style (Figs. 4H–4K and 8B–8D). In the style, each synlateral bundle branches into two, resulting in four bundles adjacent to the stylar canal (sy’) (Figs. 4L and 8E). These bundles are no longer visible at the height of the stigma (Figs. 4M and 8F)

The placental complex (pl) extends through the ovarian septum (Figs. 4H, 4I, 8A and 8B), diverging into two placental bundles at the height of the placenta (Figs. 2B and 4J).

Dorsal bundles were not observed in the carpels.

### Pattern B: carpels vascularized by synlateral and dorsal bundles (Figs. 3B and 5)

**Figure 9:**
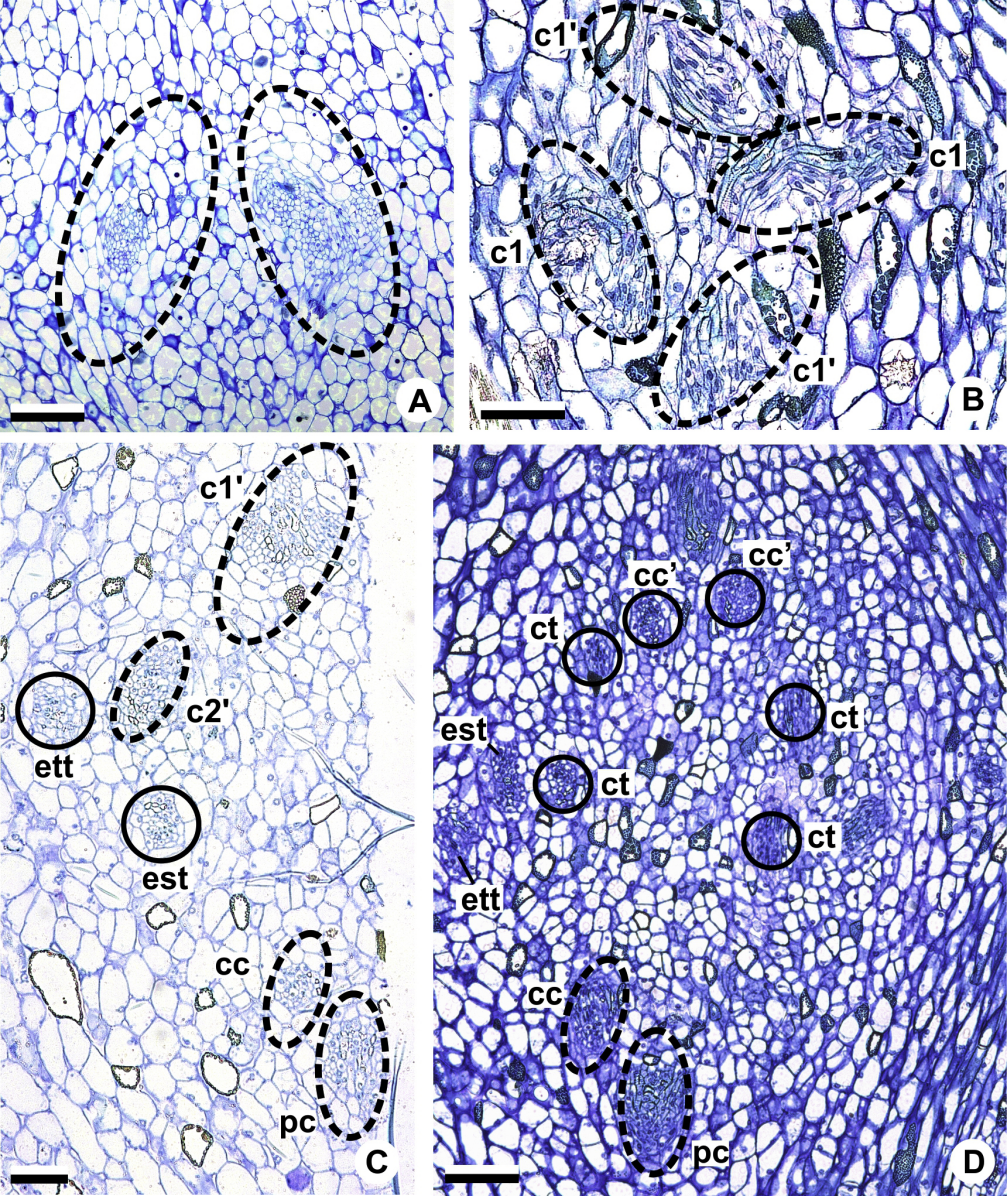
Floral vasculature of *A*. *affine* (*A*. sect. *Pachyneurium* series *Pachyneurium*)** (A, C, D) and *A. scandens* (*A*. sect. *Tetraspermium*) (B), in transverse sections of flowers at female anthesis. (A) Axis of the spadix with two vascular complexes. (B) Floral base showing the formation of four vascular complexes. (C) Floral base showing divergence of external stamen and tepal traces. (D) Floral base showing the divergence of both carpellary and vascular traces. c1’, internal vascular complex; c2’, vascular complex originated from c1; cc, carpellary complex; cc’, vascular trace; ct, carpellary trace; est, external stamen trace; ett, external tepal trace; pc, peripheral complex. Scale Bars: A = 200 µm; B, C = 100 µm; D = 150 µm.

Pattern B was observed in three of the studied species: *A. affine* (*A*. sect. *Pachyneurium* series *Pachyneurium*), *A. obtusum* and *A. scandens* (*A*. sect. *Tetraspermium*). As in pattern A, the vasculature of the axis of the spadix diverges to the flower, branching into two (Fig. 9A); at the base of the flower, four vascular complexes are visible, referred to here as external (c1) and internal complexes (c1’) (Figs. 5A and 9B). However, the destination differs for these two complexes, as described below.

**Figure 10:**
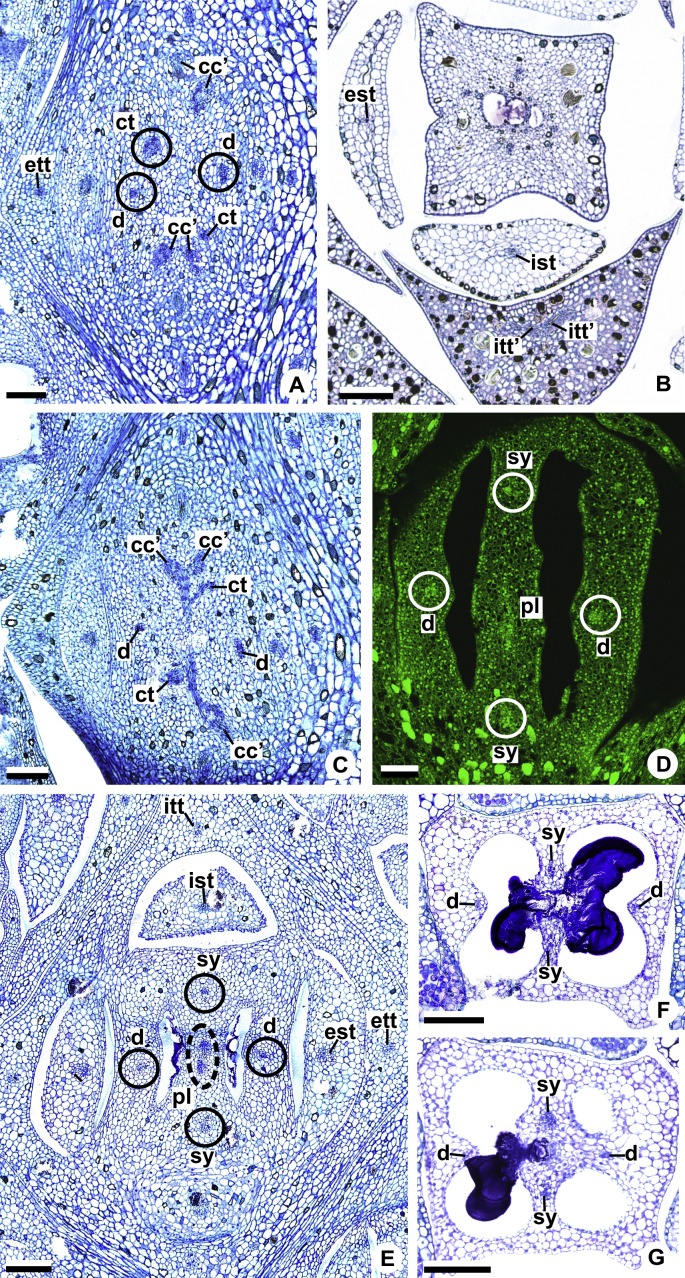
Floral vasculature of *A*. *affine* (*A*. sect. *Pachyneurium* series *Pachyneurium*) (A–C, E), *A. obtusum* (D) and *A. scandens* (F, G) (*A*. sect. *Tetraspermium*), in transverse sections of flowers. (A) Floral base with both carpellary and vascular traces. (B) Flower showing the initial branching of internal tepal trace. (C) Floral base showing the formation of carpellary supply. (D) Ovarian base with both dorsal and synlateral carpellary bundles, and placental complex (CLSM). (E) Floral base showing all floral traces. (F, G) Style with dorsal and carpellary synlateral bundles, from the height where the stylar canal opens in the ovary (F) and to the height of the apical septum (G). cc’, vascular trace; ct, carpellary trace; d, dorsal carpellary bundle; est, external stamen trace; ett, external tepal trace; ist, internal stamen trace; itt, internal tepal trace; itt’, branching of internal tepal trace; pl, placental complex; sy, synlateral carpellary bundle. Scale bars: A, C, E = 200 µm; B, D = 100 µm; F = 50 µm; G = 25 µm.

Initially, each of the two external complexes (c1) (Figs. 5A and 9B) develops two others (c2 and c2’) (Fig. 5B). Each complex (c2) diverges into two traces: one to the external tepal and the other to the external stamen (Figs. 5B, 5C, 9C and 9D). The trace of the external tepal (ett) remains singular for almost its entire length (Figs. 5C–5K and 10A), except in the upper third, where it branches into two (ett’) (Figs. 5L and 5M); the trace of the external stamen (est) extends to the connective (Figs. 5D–5J and 10B).

**Figure 11:**
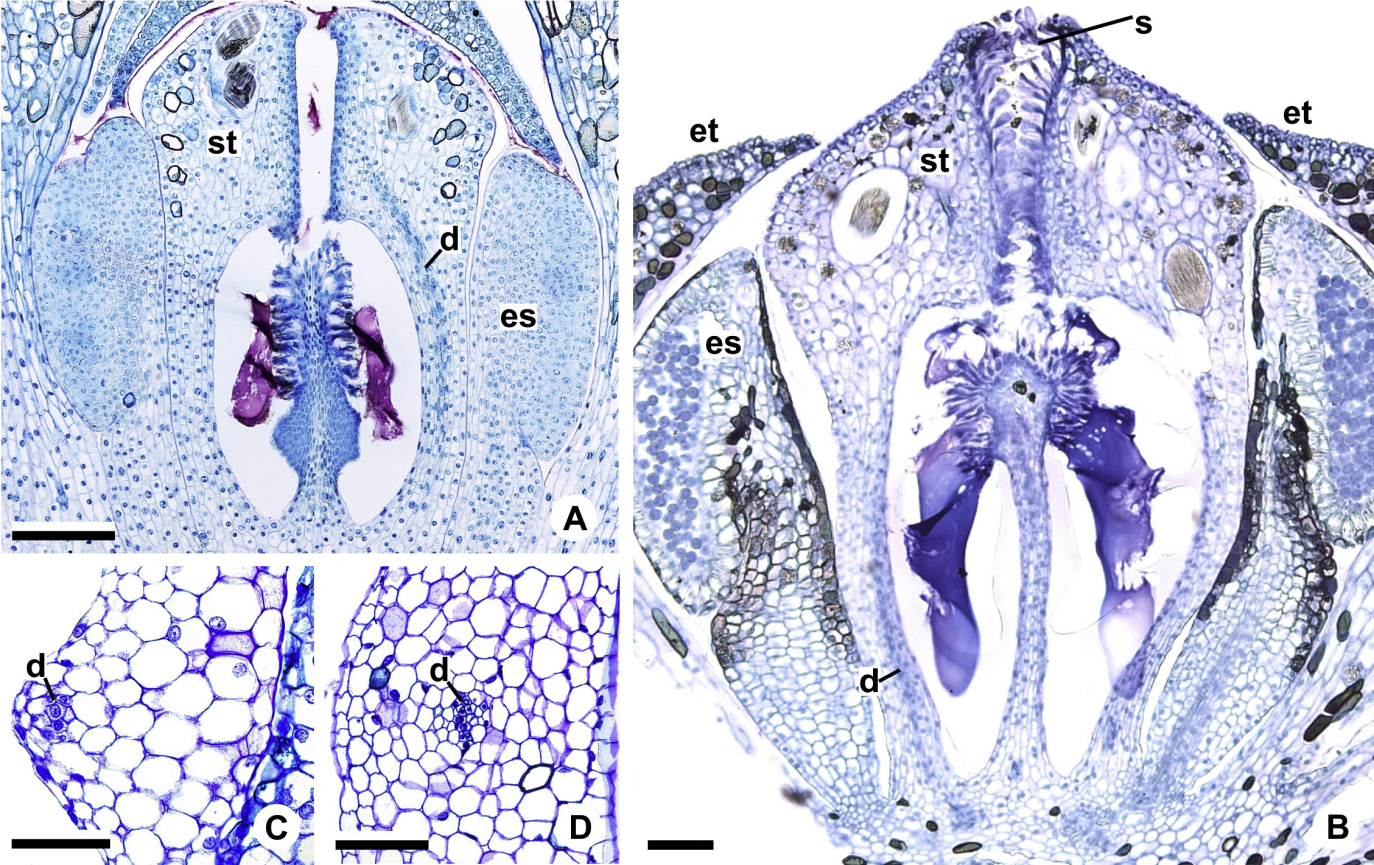
Floral vasculature of *A*. *affine* (*A*. sect. *Pachyneurium* series *Pachyneurium*) (A, D) and *A. scandens* (*A*. sect. *Tetraspermium*) (B, C). (A, B) Median longitudinal sections of developing (A) and at anthesis (B) gynoecia showing dorsal carpellary bundles. (C, D) Details, in transverse sections, of the ovarian wall at female anthesis, showing location of the dorsal carpellary bundle. d, dorsal carpellary bundle; es, external stamen; et, external tepal; s, stigma; st, style. Scale bars: A, B = 100 µm; C = 25 µm; D = 50 µm.

Each complex (c2’) diverges into two carpellary traces (ct) (Figs. 5C, 5D, 9D and 10A). One of these carpellary traces remains as the dorsal bundle of the carpel (d) (Figs. 5E–5G), and the other aids in the formation of the placental complex (pl) (Figs. 5G, 5H and 10C). Each dorsal bundle (d) extends through the median plane of each carpel until the height of the style (Figs. 3B, 5G–5K, 10D–10G, 11A and 11B), and remains adjacent to the stylar canal (Fig. 5L). In the transverse section of the carpel, it is possible to observe the variation in the location of the dorsal bundle in relation to the epidermis and the mesophyll: in *A. obtusum* and *A. scandens* (Fig. 11C), the dorsal bundle is located next to the internal epidermis; in *A. affine* (Fig. 11D), it is located in the median plane of the mesophyll, equidistant from the internal and external epidermis. The dorsal bundle of the carpel is no longer visible at the height of the stigma (Fig. 5M).

At the base of the flower, each of the other two vascular complexes (c1’) (Figs. 5A and 5B) originates two smaller complexes: one peripheral (pc) and one carpellary (cc) (Figs. 5C and 9C). Each peripheral complex (pc) (Fig. 5C) diverges into two traces: one to the internal tepal and the other to the internal stamen (Figs. 5D–5F and 9C). The trace of the internal tepal (itt) remains singular for almost its entire length (Figs. 5E–5K), except in the upper third, where it branches into two (itt’) (Figs. 5L, 5M and 10B). The trace of the internal stamen (ist) extends to the connective (Figs. 5D–5J and 10B).

Each of the two carpellary complexes (cc) (Figs. 5C, 9C and 9D) diverges into two traces (cc’) (Figs. 5D, 9D and 10A), which merge (Figs. 5E–5F), and together with the carpellary traces (ct), derived from c2’, (Figs. 5G and 10C), split into two peripheral bundles (sy) (Figs. 5H, 10D and 10E), and a placental complex (pl) (Figs. 5H, 10D and 10E). These peripheral bundles and placental complex are heterocarpellary and they are here denominated synlateral bundles. Each synlateral bundle, located between the margin of the two carpels and the ovarian wall (Figs. 10D and 10E), extends to the height of the style (Figs. 5H–5K and 10D–10G). In the style, each of these bundles remains adjacent to the stylar canal (Fig. 5L). These bundles are no longer visible at the height of the stigma (Fig. 5M).

The placental complex (pl) extends through the ovarian septum (Figs. 5H, 5I, 10D and 10E) and diverges at the height of the placenta into four placental bundles in *A. obtusum* and *A. scandens* (Figs. 2F and 5J), and into two placental bundles in *A. affine* (Figs. 2C and 11A).

## Discussion

Our results, based on the analysis of 20 species belonging to *Anthurium* sect. *Dactylophyllium*, *A*. sect. *Pachyneurium* series *Pachyneurium*, *A*. sect. *Tetraspermium* and *A*. sect. *Urospadix*, broaden the knowledge on floral vasculature of this neotropical genus of Araceae and reveal the homogeneity of this characteristic in the stamens and tepals, irrespective of the species and sections studied.

The greatest variations observed here are in relation to the carpels, corroborating the heterogeneity of the gynoecium vasculature of *Anthurium*, as reported by previous studies (Carvajal, 1977; Barabé, Forget & Chrétien, 1984). Although the vasculature of only two species of the genus has been studied previously, such results demonstrate the existence of two distinct vascular patterns. Our data allow the addition of a third vascular pattern and contribute to the characterization of the carpel of the genus in Araceae.

The first description of floral vasculature in *Anthurium* was presented by Carvajal (1977) in their study with *A. denudatum* (*A*. sect. *Belolonchium*). In this species, two ventral bundles and two dorsal bundles were observed at the base of the ovary. In the transverse section of the median plane of the ovary, only placental bundles were observed, though without knowledge of their origin, whether they were formed from ventral bundles or from dorsal bundles of the carpels. This pattern was not observed in any of the species studied in the present work.

The second description of the floral vasculature of the genus was presented by Barabé, Forget & Chrétien (1984) in their study with *Anthurium lhotzkyanum* (=*A. augustinum*) (*A*. sect. *Urospadix*). In the gynoecium of this species, two ventral complexes and two placental bundles were observed both at the base and in the median plane of the ovary. In tranverse sections at the height of the style, the ventral bundles were observed surrounding the stylar canal. There is no report of dorsal bundles of the carpels. This pattern of gynoecium vasculature was corroborated by the majority of the species we analyzed that belong to *A*. sect. *Dactylophyllium*, and in some species of *A*. sect. *Pachyneurium* series *Pachyneurium* and *A*. sect. *Urospadix*, here called as Pattern A. However, in the present study another terminology is proposed to refer to this ventral vascular supply of the carpels which will be further discussed.

For the first time for this genus, our data show the existence of a third pattern of carpellary vasculature, called as Pattern B. In our sample, the occurrence of this pattern is restricted to only one of the studied species of *A*. sect. *Pachyneurium* series *Pachyneurium* (*A. affine*) and to all of the studied species of *A*. sect. *Tetraspermium* (*A. obtusum* and *A. scandens*). These three species all have carpels vascularized by both synlateral and dorsal bundles.

In angiosperms, each carpel is generally vascularized by three bundles: one follows along its median plane, called dorsal or median bundle; and the other two continue along its margins, called lateral or ventral bundles (e.g., Eames, 1931; Eames, 1951; Puri, 1951; Eyde, 1971; Eyde, 1975; Endress, 1994; Leins & Erbar, 2010). This pattern of carpellary vasculature may vary with lateral or additional bundles described in many taxa, as basal angiosperms (e.g., Igersheim & Endress, 1998), some monocots as Acorales (e.g., Buzgo & Endress, 2000), Alismatales (e.g., Igersheim, Buzgo & Endress, 2000; Remizowa et al., 2011), Commelinales (e.g., Hardy & Stevenson, 2000; Hardy, Stevenson & Kiss, 2000), Poales (e.g., Remizowa et al., 2012; Reynders et al., 2012) and Zingiberales (e.g., Box & Rudall, 2006), and also amongst eudicots (e.g., Litt & Stevenson, 2003; Nuraliev, Sokoloff & Oskolski, 2011).

Within Alismatales, Araceae present some representatives, as *Pothos* (Eyde, Nicolson & Sherwin, 1967; Buzgo, 2001), *Anthurium denudatum* (Carvajal, 1977), *Lysichiton camtschatcensis* (L.) Schott (Barabé & Labrecque, 1984), *Spathiphyllum wallisii* Regel (Barabé & Chrétien, 1986), and *Zamioculcas zamiifolia* (Lodd.) Engl. (Barabé & Forget, 1988), in which the carpels are vascularized by dorsal bundles and by other bundles that run along their margins. In the ovary these latter bundles are located on the opposite side of the ovarian septum and have been interpreted as pertaining to adjacent carpels (Eyde, Nicolson & Sherwin, 1967; Barabé & Labrecque, 1984; Barabé, Forget & Chrétien, 1984; Barabé & Chrétien, 1986; Igersheim, Buzgo & Endress, 2000; Buzgo & Endress, 2000). Although there is a consensus on the nature of the carpellar bundles, distinct terminologies have been used to refer to them, for exafmple: ventral bundle complex (Barabé & Labrecque, 1984; Barabé, Forget & Chrétien, 1984; Barabé & Chrétien, 1986), ventral bundle (Carvajal, 1977; Barabé & Forget, 1988), synlateral bundle (Igersheim, Buzgo & Endress, 2000), and septal vascular bundle (Buzgo, 2001). This vascular supply may branch out at the height of the style, forming two other ventral bundles (Barabé & Labrecque, 1984; Barabé, Forget & Chrétien, 1986; Barabé & Chrétien, 1986).

In the species studied of *Anthurium*, the characteristics of the bundles to vascularize the margins of the carpels, either branching (Pattern A) or not (Pattern B) in the style, suggest that they resemble (in position and function) those bundles located opposite the ovarian septum described in previous studies. *Anthurium* also presents a set of vascular bundles located in the ovarian septum that derive the traces that supply the ovules. In previous studies, this vascular supply is referred to as ventral bundle (Carvajal, 1977) or placental column (Barabé, Forget & Chrétien, 1984) and in the present study, as a placental complex. Our data reveal that this placental complex has the same origin as the bundles located on the margins of the carpels, deriving differently from distinct complexes in Patterns A and B.

The common origin of the bundles of the margins of the carpels and of the ovarian septum suggests the use of a single term to denominate this carpellary supply. Given the different terminologies found in the literature, in the present study we chose to denominate the bundles and placental complex as synlateral bundles, since the traces from which they derive come from different carpels. This term is in accordance with the previous study by Igersheim, Buzgo & Endress (2000) in representatives of basal monocotyledons, including Araceae.

In *Acorus* (Acoraceae, Alismatales), a genus previously included as early-divergent in Araceae, carpels are supplied by vascular bundles of a central column (Buzgo & Endress, 2000). Thus, the absence of the dorsal bundles is compensated by the transference of its function to the vascular bundles of central column, which vascularizes the ovules and branch supplying the style (Buzgo & Endress, 2000); in other words, the loss of the vascular tissues during floral development leads to a reorganization of the existing bundles for the supply to the floral organs.

On the other hand, in species of *Anthurium* which gynoecia lack dorsal bundles (Barabé, Forget & Chrétien, 1984; described in the present study as Pattern A), their absence is compensated by splitting the synlateral bundles. These synlateral bundles branch into four bundles (sy’) adjacent to the stylar canal, suggesting their equivalence (in position and function) to the dorsal bundles observed in *A. affine* (*A*. sect. *Pachyneurium* series *Pachyneurium*), *A. obtusum* and *A. scandens* (*A*. sect. *Tetraspermium*), also studied in the present work.

Also in relation to the floral structure, another highlight regarding the heterogeneity of the carpellary vasculature is the variation in the location of the synlateral bundles. In the majority of the species studied here, the synlateral bundles are located between the margin of the carpel and the ovarian wall. However, only in *A. obtusum* and *A. scandens* (*A*. sect. *Tetraspermium*) are the synlateral bundles located in the ovarian wall. This variation in the location of the synlateral bundles in *A*. sect. *Tetraspermium* may be related to the supply to the apical and ovarian septa found in this group, in addition to the existence of two ovules per locule. The apical septum was reported for *A*. *scandens* by Poli, Temponi & Coan (2015) as being related to the ovary’s reduced size to accommodate the ovules.

**Figure 12:**
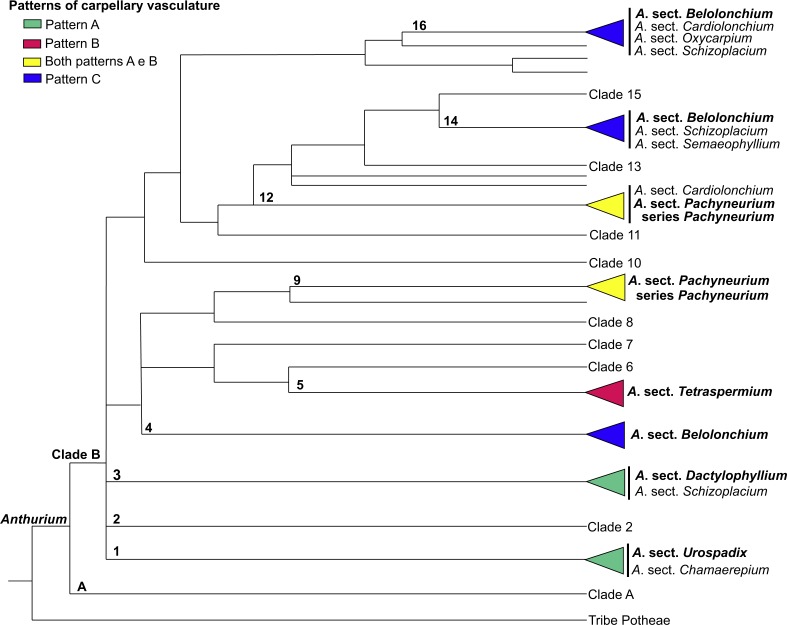
Phylogenetic tree of *Anthurium* (adapted from Carlsen & Croat, 2013) showing the distribution of the three patterns of carpellary vasculature. Sections with available carpellary vasculature data are indicated in bold.

More important than highlighting the existence of these three patterns of carpellary vasculature in *Anthurium* is to emphasize the variation in terms of the origin of the dorsal and synlateral bundles. Our results provide the first evidence that the carpellary bundles in *Anthurium* possess a mixed nature; they originate from two distinct vascular complexes and present a relationship intrinsic to the branching, or not, of the external complex (referred to here as c1) of the floral base into an additional complex, referred to here as c2.

The results presented here, together with those already reported, show that the vasculature of the carpels in *Anthurium* follow three main patterns: Pattern A, in which the gynoecium is vascularized only by synlateral bundles, as verified in the majority of the species of *A*. sect. *Dactylophyllium, A*. sect. *Pachyneurium* series *Pachyneurium* and *A*.sect. *Urospadix* (Barabé, Forget & Chrétien, 1984; Fig. 12); Pattern B, in which the gynoecium is vascularized by synlateral and dorsal bundles, as observed in *A. affine*, a species belonging to *A*. sect. *Pachyneurium* series *Pachyneurium*, and in *A*. *scandens*—*A*. sect. *Tetraspermium* (Fig. 12); and Pattern C, in which the gynoecium is vascularized only by ventral bundles (here interpreted as synlateral bundles), while the dorsal bundles are vestigial (Carvajal, 1977), as reported for a single species of *A*. sect. *Belolonchium* (Fig. 12).

While analyzing the patterns of vasculature described here, and the possible use of the vascular characteristics for better delimitation of the sections of *Anthurium*, we noted that the presence of an apical septum and carpels vascularized by dorsal and synlateral bundles (Pattern B)—observed in all of the studied species of *A*. sect. *Tetraspermium* (50% of all the species of this group)—might represent synapomorphies of this section.

In the evolutionary hypothesis presented in the Bayesian analysis by Carlsen & Croat (2013) (see adapted Fig. 12 in the present study), *Anthurium* is divided into Clades A and B that contain the large majority of the species. The three patterns of carpel vasculature occur in Clade B and deserve to be investigated in a larger number of species, particularly from *A*. sect. *Pachyneurium,* since this section presents this carpel variation and was already designated as being more than one group of species, both by works of classical taxonomy (Croat, 1991) as well as by the phylogenic study of the genus, conducted by Carlsen & Croat (2013). A possibility is that Pattern A could be a plesiomorphy for all *Anthurium* species, given that it is also found in other species of *A*. series *Pachyneurium*, and that the appearance of dorsal bundles is derived within the genus. However, the lack of samples from other sections precludes a clear distinction between these two patterns and reinforces the importance of vasculature data in the genus.

## Additional Information and Declarations

### Field Study Permissions

The following information was supplied relating to field study approvals (i.e., approving body and any reference numbers): The material examined is listed in Table 2, based on the following field permits: SisBio permanent permit no. 40816-2 (April 2011 to present) to LP Poli; SisBio collecting permits no. 28776 (June 2011–May 2014) and 28686 (June 2011–May 2014) to LG Temponi.

### Data Availability

The following information was supplied regarding data availability: The raw data is included in Table 2.
